# Endoscopic transpapillary stent placement in patients with necrotizing pancreatitis and disconnected main pancreatic duct syndrome

**DOI:** 10.3389/fsurg.2023.1328304

**Published:** 2023-12-11

**Authors:** Aleksey V. Shabunin, Zurab A. Bagatelia, Vladimir V. Bedin, Irina Yu Korzheva, Dmitry V. Shikov, Andrei A. Kolotilshchikov, Elena A. Kalashnikova, Serghei Covantsev

**Affiliations:** ^1^ Department of Surgery, № 76, Botkin Hospital, Moscow, Russia; ^2^ Department of Clinical Research and Development, Botkin Hospital, Moscow, Russia; ^3^ Department of Surgery, Russian Medical Academy of Continuous Professional Education, Moscow, Russia; ^4^ Department of Endoscopy, Botkin Hospital, Moscow, Russia; ^5^ Department of Endoscopy, Russian Medical Academy of Continuous Professional Education, Moscow, Russia

**Keywords:** acute pancreatitis, necrotizing pancreatitis, main pancreatic duct, disconnected main pancreatic duct syndrome, endoscopic stent placement, endoscopic retrograde cholangiopancreatography

## Abstract

**Introduction:**

Pancreatic necrosis is one of the most severe acute abdominal conditions, accounting for 15%–20% of all patients with acute pancreatitis and characterized by significant rates of postoperative complications and mortality. Patients with pancreatic necrosis, in which pathological changes are localized in the proximal pancreas and retroperitoneal space, deserve special attention. This form of the disease includes patients with disconnected main pancreatic duct (MPD) syndrome who have a difficult prognosis.

**Aim:**

The aim of the study was an improvement of treatment results in patients with necrotizing pancreatitis and signs of the dissociation of the pancreas duct system using the endoscopic transpapillary stent placement method.

**Material and methods:**

This study was a retrospective cohort study. There were 32 patients with acute necrotizing pancreatitis who were managed using the endoscopic transpapillary stent placement method between 2019 and 2021. Disconnected MPD syndrome was diagnosed in all 32 patients. In total, 26 patients were admitted to hospital in the first 72 h, while 6 patients were admitted after 72 h. We diagnosed the necrotizing process located in the proximal and central areas of the pancreas and peripancreatic space in all these patients (“model III”).

**Results:**

Positive results related to transpapillary stent placement were noted in 24 (75%) patients (first cohort). A total of 20 patients from this group were admitted to hospital in the first 48 h, and 4 patients were admitted later than 72 h from the onset of disease. Moreover, 8 patients (25%; second cohort) failed to succeed in transpapillary stent placement. Complications in the first cohort occurred in 3 (12.5%) patients: dislocation of the stent into the duodenum occurred in 1 patient, and bleeding after papillosphincterotomy took place in 2 patients. Meanwhile, infected necrotized pancreatitis developed in 5 patients, and 1 patient (5%) died. Complications among the second cohort occurred in 2 (25%) patients: erosive bleeding (after debridement). Infected necrotized pancreatitis developed in 4 patients, and 2 patients (25%) died.

**Conclusions:**

Endoscopic transpapillary stent placement is an effective minimally invasive approach in the management of patients with necrotizing pancreatitis.

## Introduction

Pancreatic necrosis is one of the most severe acute abdominal conditions, accounting for 15%–20% of all patients with acute pancreatitis and characterized by significant rates of postoperative complications and mortality (which can go up to 39%–45% in case of infection) (1–4).

In recent years, significant progress has been made in the diagnosis and treatment of pancreatic necrosis, associated with the use of modern instrumental diagnostic methods, a multidisciplinary approach, the improvement of intensive care, and the introduction of low-traumatic methods at the stage of surgical treatment (5). This is all reflected worldwide in national and international clinical guidelines (6).

Patients with pancreatic necrosis, in whom pathological changes are localized in the proximal pancreas and retroperitoneal space, deserve special attention. This form of the disease includes patients with disconnected main pancreatic duct (MPD) syndrome (7, 8).

Disconnected pancreatic duct syndrome is a condition that is characterized by a violation of the discharge of pancreatic juice into the duodenum due to the separation of the proximal and distal parts of the MPD. The process of dissociation of the ductal system occurs at the level of necrosis of the head and isthmus of the pancreas, which subsequently leads to the formation of a pronounced parapancreatic infiltrate due to the aggressive effect of the pancreatic juice on the surrounding tissues of the retroperitoneal space (8).

A diagnosis of dissociation of the MPD is based on data using computed tomography (CT) with intravenous contrast, and in some cases, on additional data obtained during magnetic resonance imaging (MRI), endoscopic ultrasonography (EUS), and endoscopic retrograde cholangiopancreatography (ERCP) (9).

Currently, there are studies in the literature that describe the technique of transpapillary stenting of the MPD in the treatment of patients with acute pancreatitis (10), but do not describe clear indications for its use nor indicate the optimal timing of the intervention. In addition, the question of the duration of the stent remains relevant.

The aim of this study was to explore an improvement of treatment results in patients with necrotizing pancreatitis and signs of the dissociation of the pancreas duct system using the endoscopic transpapillary stent placement method.

## Materials and methods

This was a retrospective analysis of the treatment results of 32 patients with pancreatic necrosis at the surgical clinic of Botkin Hospital in the period between 2019 and 2021, and who underwent transpapillary stenting of the MPD due to its disconnection. All patients underwent CT of the abdominal organs with intravenous contrast enhancement within 24–48 h from the onset of the disease or immediately upon admission to the hospital. All patients underwent laboratory testing (leukocytes, amylase, aspartate aminotransferase (AST), alanine aminotransferase (ALT), total and direct bilirubin) and CT with intravenous contrast every 7–10 days or in case of deterioration of the patient's condition. All patients received standard care including antibiotic treatment (piperacillin/tazobactam 3 × 4.5 g per 24 h IV), spasmolytic drugs (papaverine 40 mg/platyphylline 2 mg IV solution), and octreotide (0.3 ml intradermal).

**Figure 1:**
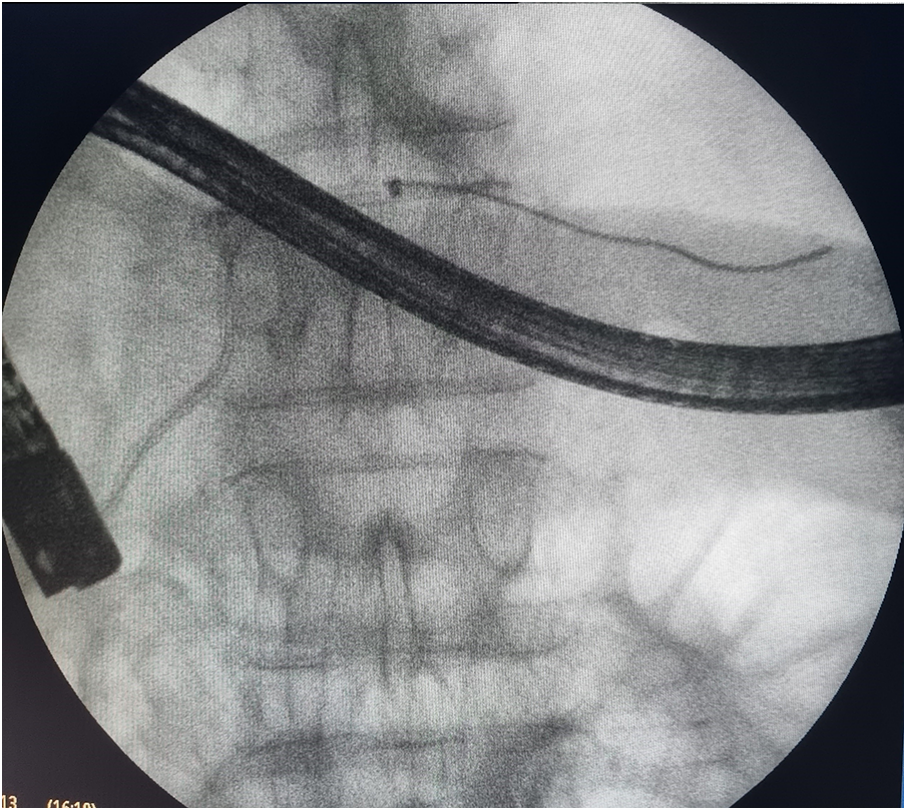
Endoscopic retrograde cholangiopancreatography: endoscopic transpapillary stent placement of the main pancreatic duct.

The surgical clinic of Botkin Hospital has developed the principle of diagnostic modeling of pancreatic necrosis, based on accurate clinical, instrumental, and morphological diagnostics, which allows all patients to be divided into four “models” depending on the location and extent of necrotic changes in the pancreatic parenchyma and retroperitoneal tissue (Table 1). Necrotic changes localized in the parenchyma of the pancreatic head and its isthmus, as well as an infiltrative lesion of the right retroperitoneal space, characteristic of the “model III,” were diagnosed among all of the patients who underwent stenting (Figure 1).

**Table 1:** Pancreatic necrosis models used for assessment.

Model	Imaging	Description
I	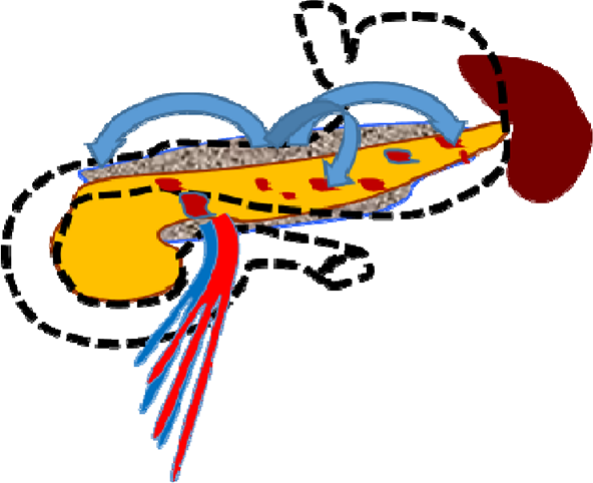	Small focal necrosis of the pancreas, parapancreatic infiltrate
II	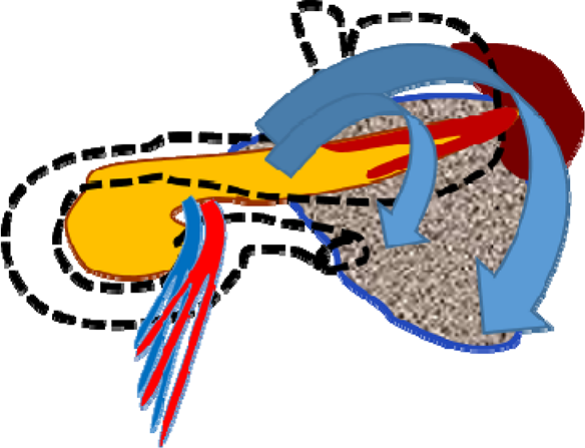	Necrosis of the distal pancreas, infiltration of retroperitoneal tissue “left-sided type”
III	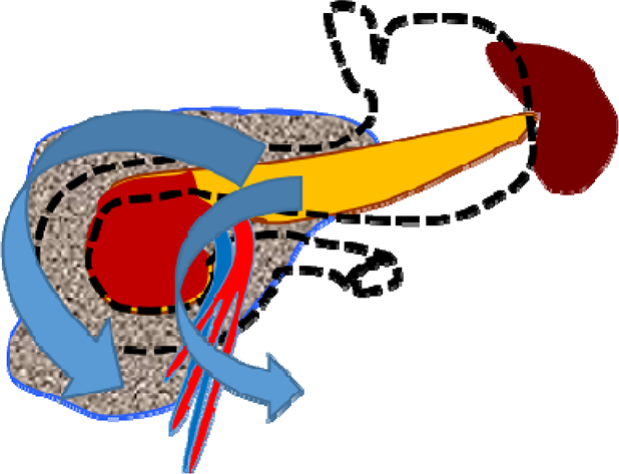	Necrosis of the proximal pancreas, retroperitoneal tissue infiltration “right-sided type”
IV	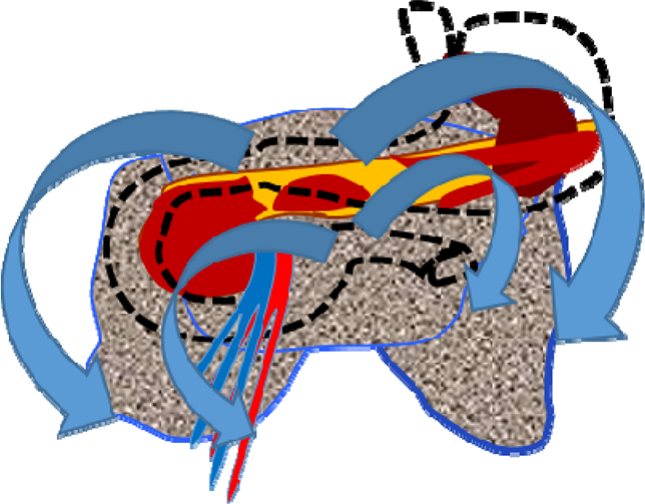	Large focal necrosis of the pancreas, infiltration of retroperitoneal tissue in the proximal, central, distal sections “mixed type”

We defined dissociated main pancreatic duct syndrome as total disruption of the MPD visible at CT, which corresponded to model III of the Botkin Classification of Pancreatic Necrosis (Table 1). Pancreatic stenting was performed in case necrosis of the parenchyma was more than 30%. The diagnosis was further confirmed endoscopically during the procedure in cases when a guidewire could not traverse the point of disconnection, with non-opacification of the pancreatic duct upstream from the site of disruption.

Technical success meant successful stenting when active drainage of the pancreatic juice could be achieved. Clinical success and failure were assessed in terms of clinical and laboratory response.

MPD was stented under X-ray operating room conditions, under combined endotracheal anesthesia, using endoscopic ultrasonography navigation, video duodenoscopy, and ERCP. A plastic multiperforated stent (5–7 Fr in diameter) was used to correct the disconnection of the pancreatic ductal system. All patients were managed according to the step-up approach (11, 12). Pancreatic stenting was performed in an attempt to disrupt the vicious cycle of edema and inflammation.

The patients were divided into two groups (cohorts) depending on the result obtained after the endoscopic intervention. The first cohort consisted of 24 patients in whom the attempt to perform stenting of the MPD was successful. The second cohort consisted of 8 patients in whom pancreatic stenting was unsuccessful. All patients were managed with a similar therapeutic approach.

The characteristics of the patients were assessed using the Wilcoxon test for abnormally distributed continuous data and Fisher’s exact test for categorical data. A *p*-value <0.05 was considered statistically significant.

## Results

In total, 27 (84.3%) patients were admitted within the first 72 h from the onset of the disease, and the remaining 5 (15.7%) patients were admitted more than 72 h after the onset of the disease. According to the results of the survey, the indications for stenting of the main pancreatic duct were set in all of these patients (Figure 1).

The groups were comparable in terms of sex (16 men and 8 women in the first cohort; 5 men and 3 women in the second cohort), age (mean age in the first cohort 42.55 ± 4.15 years; mean age in the second cohort 46 ± 7.65 years), biochemical changes, and pain intensity (Table 2).

**Table 2:** Comparison of the groups.

	Successful stenting (first cohort, *n* = 24)	Unsuccessful stenting (second cohort, *n* = 8)	*p*
Age (years)	42.55 ± 4.15	46.00 ± 7.65	>0.05
Sex (M/F)	16/8	5/3	>0.05
Leukocytes (10^9^)	13.12 ± 6.90	14.62 ± 4.8	>0.05
Amylase (IU/L)	1146.54 ± 1770.46	1050.10 ± 1824.46	>0.05
Total bilirubin (mmol/L)	51.15 ± 49.98	49.26 ± 47.62	>0.05
Direct bilirubin (mmol/L)	23.73 ± 32.29	24.08 ± 30.25	>0.05
ALT (IU/L)	107.76 ± 128.76	102.41 ± 132.44	>0.05
AST (IU/L)	96.89 ± 82.91	98.77 ± 84.12	>0.05
VAS (points)	7.6 ± 1.5	7.5 ± 1.2	>0.05
Hospital stay (days)	40.2 ± 3.5	54.6 ± 4.5	<0.05

**Figure 2:**
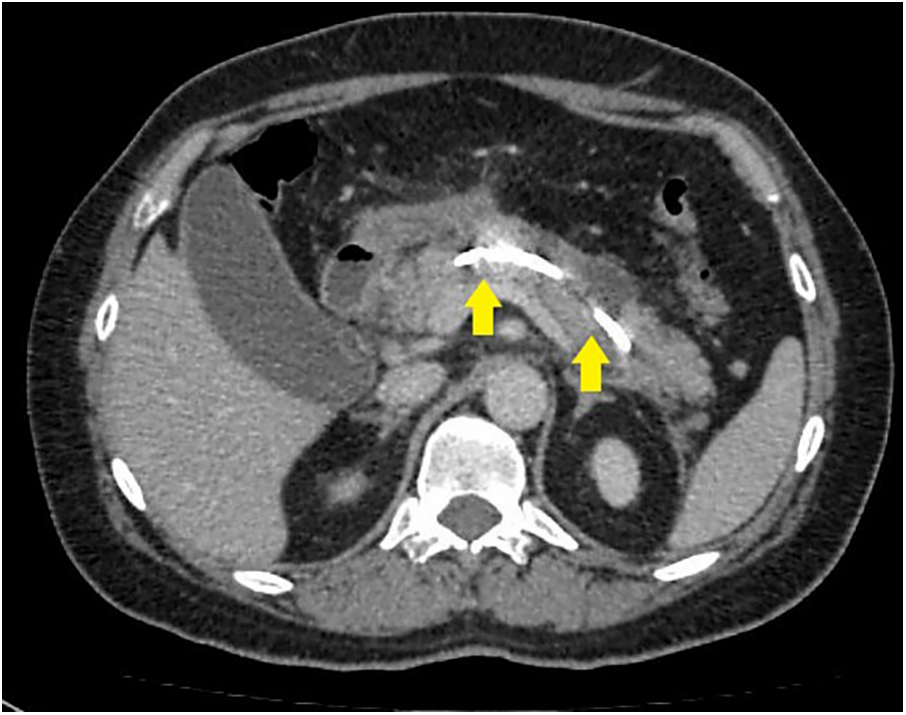
CT image after endoscopic transpapillary stent placement in the main pancreatic duct in patients with necrotizing pancreatitis (stents in the main pancreatic duct and common hepatic duct are indicated by arrows).

Successful stent placement (Figure 2) was possible in 24 (75%) patients (first cohort), 20 of whom were hospitalized within 48 h from the onset of the disease; 4 patients were hospitalized 48–72 h from the onset of the disease. Dissociation of the MPD with leakage of contrast into the retroperitoneal space in the projection of the isthmus of the pancreas, detected during ERCP, was diagnosed in patients who were hospitalized 48–72 h from the onset of the disease, which indicates the unconditional importance of the timely implementation of a low-traumatic endoscopic stenting intervention.

**Figure 3:**
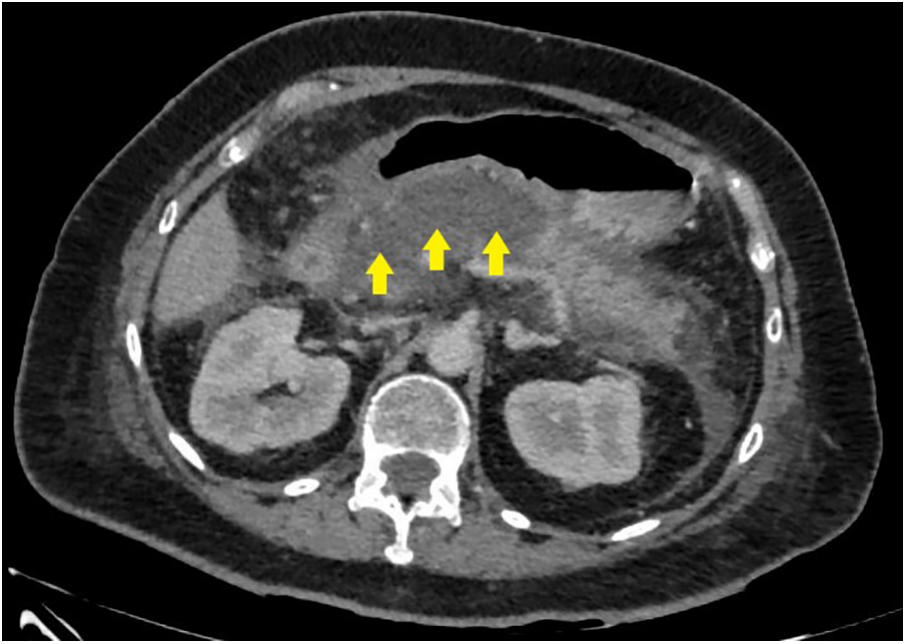
CT image before endoscopic transpapillary stent placement in the main pancreatic duct in patients with necrotizing pancreatitis (stents in the main pancreatic duct and common hepatic duct are indicated by arrows).

In 8 (25%) patients (second cohort) who failed to undergo endoscopic stenting (Figure 3) of the MPD, the study was completed by placing a nasointestinal feeding tube behind the ligament of Treitz. A total of 2 patients were admitted 48–72 h from the onset of the disease and 6 patients after 72 h.

**Figure 4:**
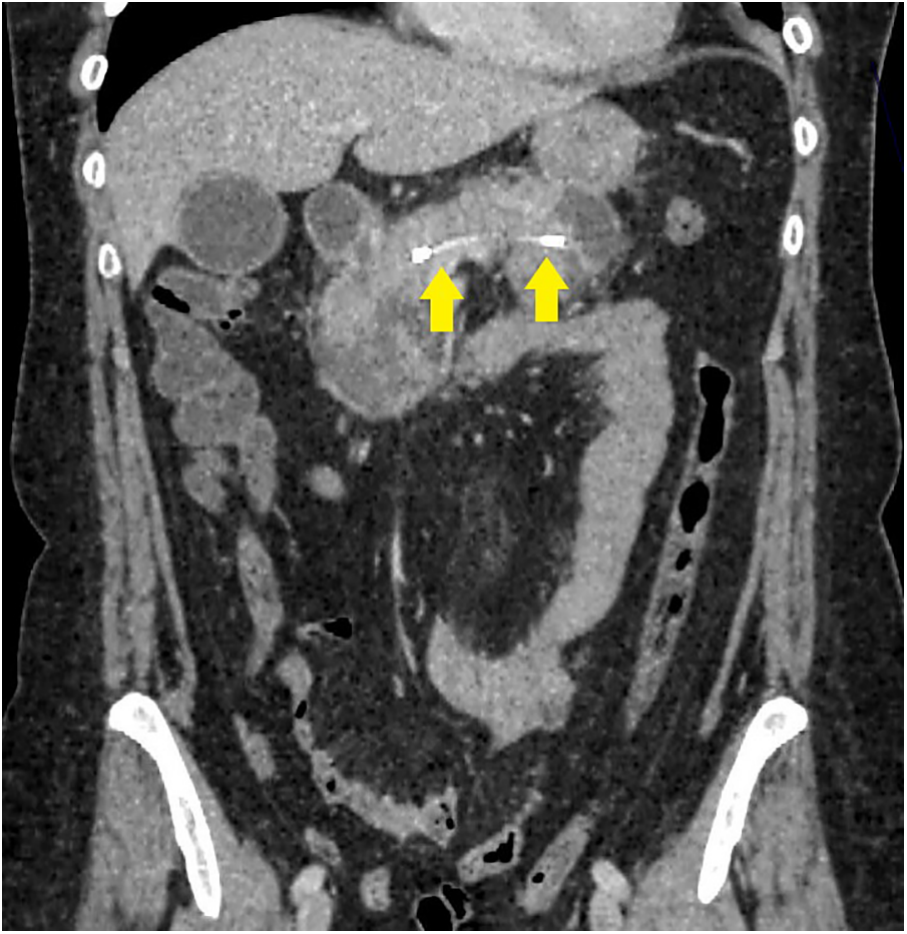
Patient with successful MPD stenting 48 h after stenting.

Among 19 (79.2%) patients of the first cohort who underwent endoscopic stenting of the pancreatic ductal system, over the next 48 h, there was a decrease in leukocytosis and a decrease in the degree of severity (and in some cases, complete relief) of abdominal pain syndrome (Table 3). According to the results of the control instrumental studies (CT with intravenous contrast enhancement), there was no further progression and increase in infiltrative-inflammatory changes in the pancreatic parenchyma and retroperitoneal tissue (Figure 4). In the remaining 5 (20.8%) patients of this group, the disease was characterized by a more stable course and a lesser degree of necrotic changes (Table 3).

**Table 3:** Comparison of the efficiency of stenting of MPD in the main group (48 h after stenting).

	Before stenting	After stenting	*p*
Leukocytes (10^9^)	13.12 ± 6.90	10.26 ± 5.63	0.04
Amylase (IU/L)	1146.54 ± 1770.46	444.33 ± 423.57	0.02
Total bilirubin (mmol/L)	51.15 ± 49.98	33.58 ± 23.16	0.01
Direct bilirubin (mmol/L)	23.73 ± 32.29	15.20 ± 14.69	0.07
ALT (IU/L)	107.76 ± 128.76	80.05 ± 84.58	0.15
AST (IU/L)	96.89 ± 82.91	74.75 ± 59.10	0.18
VAS (points)	7.60 ± 1.50	3.20 ± 1.20	0.03

Minimally invasive treatment was performed in case of infection in areas of necrosis and parapancreatic infiltrate. Minimally invasive treatment was performed in 5 (20.8%) patients from the first cohort: 1 patient underwent ultrasonography-guided drainage, 1 patient underwent endoscopic transluminal sequestrectomy (endoscopic sanitation was performed three times), and 3 patients underwent a combination of the above-mentioned methods.

In the first cohort, the development of the following complications was noted: 1 patient experienced dislocation of the pancreatic stent into the lumen of the duodenum (endoscopic removal and re-installation); and 2 patients had bleeding from the area of the performed endoscopic papillosphincterotomy (both underwent combined endoscopic hemostasis (12.5% in total)). One patient (5%) died due to the progression of multisystem organ failure induced by pancreatogenic shock.

**Figure 5:**
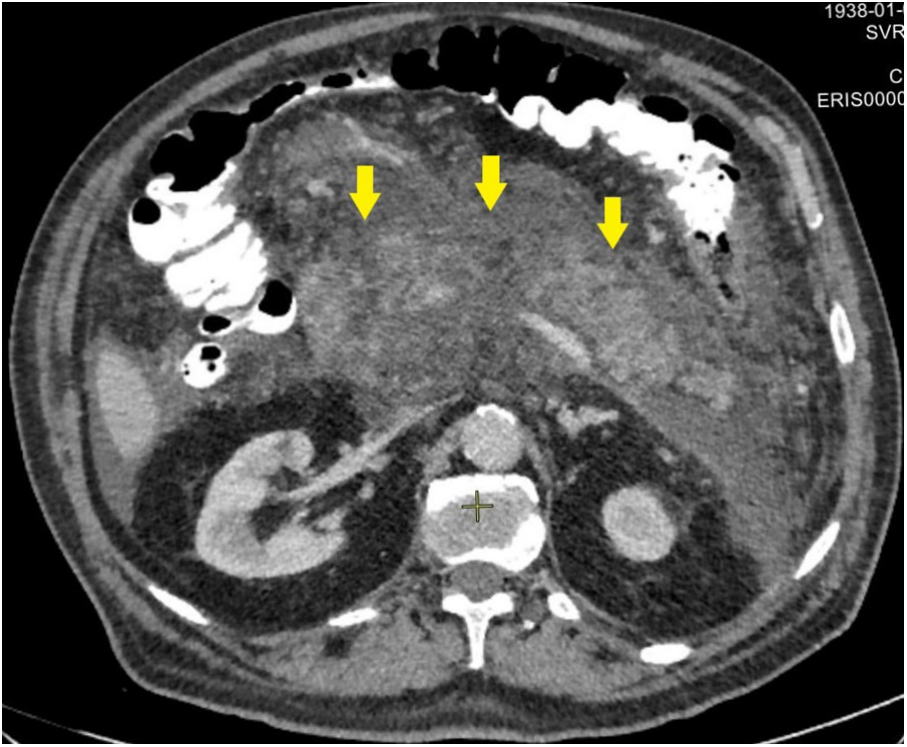
CT image of necrotizing pancreatitis in patients, who failed in endoscopic transpapillary stent placement of the main pancreatic duct (peripancreatic infiltrate is indicated by arrows), 72 h after a failed attempt.

Patients who did not achieve technical success when attempting to stent the MPD (second cohort) were characterized by significantly more pronounced infiltrative-inflammatory changes in the pancreatic parenchyma and surrounding tissue (Figure 5), as well as a severe course of the diseases (with severe intoxication and abdominal pain, a gradual increase in the prevalence of parapancreatic infiltrate).

The development of infected pancreatic necrosis in the second cohort was subsequently noted in 4 (50%) patient, which was an indication for sequestrectomy (in 1 patient, we performed an endoscopic transluminal sequestrectomy, and in 3 patients, a combination of percutaneous drainage and endoscopic transluminal sequestrectomy).

Complications were noted in 2 (25%) patients from the second cohort; both patients had arrosive bleeding at the stage of sequestrectomy. Moreover, 2 patients (25%) died due to the progression of multiple organ failure (patients were admitted later than 72 h from the onset of the disease; in both cases, endoscopic stenting attempts of the MPD were unsuccessful).

The mean hospital stay was lower in the main group (40.2 ± 3.5 days) than in the control group (54.6 ± 4.5 days).

## Discussion

Acute pancreatitis is a complicated condition associated with multiple complications (2–4). There are many areas in the management of acute pancreatitis for which no consensus exists (13). There is an ongoing discussion of the efficiency of transpapillary and transmural stenting in patients with disconnected pancreatic duct syndrome (14). There are currently no universally accepted guidelines for the diagnosis and treatment of disconnected pancreatic duct syndrome, and because the condition is underrecognized, the diagnosis is often delayed (15). However, pancreatic stenting is now a more widely performed procedure in cases of intraductal hypertension, bypass obstructing stones, restoring lumen patency in cases with dominant, symptomatic strictures, and so on (16). Therapeutic stent placement is intended to restore the proper flow of pancreatic secretion and has demonstrated positive results in several studies of early-stage pancreatitis (17–19).

In recent years, there has been a constant development and improvement of medical technologies and, in particular, modern methods of instrumental diagnostics, which makes it possible to increasingly use various low-traumatic methods of surgical treatment. Minimally invasive methods of sequestrectomy are used in patients with severe acute pancreatitis in the phase of purulent-necrotic complications. In the early phase of the development of the disease, the endoscopic transpapillary stenting technique of the pancreatic ductal system began to be increasingly used .

It is worth paying special attention to the aspects of the pathogenesis of acute pancreatitis and pancreatic necrosis: the main inflammatory and necrotic changes in the pancreatic parenchyma develop during the first 72 h from the onset of the disease. Further necrotic transformation of the surrounding retroperitoneal tissue is progressive, which leads to a deterioration in the patient's condition (20).

Our experience allows us to state that an early and timely diagnosis of the necrotic process and the reasonable use of the technique of endoscopic transpapillary stenting of the MPD, according to strict indications, can achieve positive results in the treatment of patients with severe acute pancreatitis.

The use of the described technique in patients from the first cohort led, in a significant number of cases (79.2%), to a decrease in the manifestations of the systemic inflammation, a decrease in the severity of abdominal pain syndrome, and also (which is even more significant) an interruption of further progression and an increase of infiltrative changes in the parenchyma pancreas and retroperitoneal fat, which determined the severity and prognosis of the course of the disease.

The development of an infected form of pancreatic necrosis in patients who managed to perform stenting of the main pancreatic duct was observed in fewer cases (20.8%) compared to the second cohort (50%). In addition, there was a decrease in the mean length of hospital stay from 54.6 ± 0.5 days to 40.2 ± 0.5 days and mortality. However, the reasons for failed MPD stenting are debatable especially taking into account developmental variations of the pancreatic ductal system (21).

The specified therapeutic tactics in patients with pancreatic necrosis at the initial stages of the pathological process allow for the interruption of disease progression and the development of parapancreatic infiltrate. Therefore, surgeons have the opportunity to avoid the need to act as a “catch-up” in the treatment of a formidable disease.

However, further accumulation of experience in the application of this promising endoscopic method of treatment is required. Another debatable issue that requires discussion is the clarification of the optimal timing for which a stent should be installed in the MPD. The attempt to perform an early CT scan (<72 h) in our study is to have the possibility to diagnose MPD disconnection as soon as possible.

We would like to draw special attention to the fact that such endoscopic manipulations should be performed in specialized surgical centers with relevant accumulated experience and a high degree of preparedness of medical personnel, as well as modern technical equipment, which is primarily due to the complexity of the endoscopic intervention. It is debatable whether duodenal microflora can contribute to the development of pancreatic infection. Animal models demonstrate that the small intestine is the main source of infection rather than the colon (22). Some studies underlined duodenal dysregulation as a potential risk for infection in patients with severe acute pancreatitis (23). Similarly, there are data that patients with acute pancreatitis have duodenal barrier dysfunction (24). Loss of barrier function is a risk factor for flora translocation into the inflamed tissue. The main aim of the present study was to perform MPD stenting in the earliest stage of the disease when there is time to break the vicious cycle. The evolution of pancreatic necrosis often takes several days, and it is often uncertain whether the particularly suspicious area on an imaging study is necrotic (25). Therefore, the first CT scan in our study was performed as a “set” point for further analysis.

Further active implementation of endoscopic stenting of the main pancreatic duct, according to strict indications, can lead to improved treatment outcomes in patients with severe acute pancreatitis.

The main limitations of the study include the small number of cases and assessing the patient with a standard laboratory evaluation.

## Conclusions

The use of the transpapillary endoscopic stenting technique of the main pancreatic duct in patients with pancreatic necrosis is advisable to detect pathological changes localized in the head and isthmus of the pancreas with signs of dissociations of its ductal system (“model III” of pancreatic necrosis), in the early stages from the onset of the disease (the first 72 h). It is necessary to further study this problem comprehensively to gain experience and increase the reliability and significance of the results obtained. In addition, the duration of stenting of the pancreatic ductal system (stent exposure) also needs to be clarified.

## Data Availability

The raw data supporting the conclusions of this article will be made available by the authors, without undue reservation.
